# Transglutaminase 2 Stimulates Cell Proliferation and Modulates Transforming Growth Factor-Beta Signaling Pathway Independently of Epithelial–Mesenchymal Transition in Hepatocellular Carcinoma Cells

**DOI:** 10.3390/ijms26125497

**Published:** 2025-06-08

**Authors:** Hiromi Yamaguchi, Ramiro José González-Duarte, Xian-Yang Qin, Yuriko Abe, Ichiro Takada, Benjamin Charroy, Verna Cázares-Ordoñez, Shigeyuki Uno, Makoto Makishima, Mariko Esumi

**Affiliations:** 1 Division of Biochemistry, Department of Biomedical Sciences, Nihon University School of Medicine, Tokyo 173-8610, Japan; yamaguchi.hiromi20@gmail.com (H.Y.); ramiro.gonzalezd@correo.buap.mx (R.J.G.-D.); abe.yuriko@nihon-u.ac.jp (Y.A.); takada.ichiro@nihon-u.ac.jp (I.T.); charroybenjamin@gmail.com (B.C.); verna.cazares@correo.buap.mx (V.C.-O.); uno.shigeyuki@morioka-u.ac.jp (S.U.); 2 Laboratory for Cellular Function Conversion Technology, RIKEN Center for Integrative Medical Sciences, Yokohama 230-0045, Japan; xyqin@riken.jp

**Keywords:** transglutaminase 2, hepatocellular carcinoma, epithelial–mesenchymal transition, TGF-β1, PI3K-Akt signaling, Wnt/β-catenin signaling, tumor microenvironment

## Abstract

Transglutaminase 2 (TG2) is a multifunctional protein and plays a role in cancer progression. We previously identified TG2 as an early-recurrence biomarker in hepatocellular carcinoma (HCC). TG2-knockdown (shTG2) and control (shCtl) HCC cell lines were used for comparative analyses to clarify the molecular mechanisms underlying the contribution of this protein to HCC malignancy. The proliferation of shTG2 cells was slightly but significantly decreased compared with that of shCtl cells. Differential gene expression profiling based on GeneChip arrays revealed the enrichment of the PI3K-Akt signaling pathway and showed that the expression of Dickkopf-1 and -3 (DKK1 and DKK3, respectively), inhibitors and modulators of the Wnt/β-catenin signaling pathway, was increased in shTG2 cells. The expression of epithelial–mesenchymal transition (EMT)-related genes was similar in both shCtl and shTG2 cells before and after TGF-β1 treatment, even though TGF-β1 markedly upregulated TG2. Thus, TG2 may contribute to cancer malignancy via the stimulation of cell proliferation signaling, such as PI3K-Akt and Wnt/β-catenin signaling, but not EMT. This effect might be further enhanced by humoral factors such as TGF-β1 from the tumor microenvironment.

## 1. Introduction

Liver cancer is the third leading cause of cancer-related death worldwide, and hepatocellular carcinoma (HCC), which is the most common type of liver cancer, has a high mortality rate due to its frequent recurrence [1,2,3]. The mechanism of early recurrence of HCC is not well understood. We examined the characteristics of the early recurrence of HCC by performing HCC recurrence-related proteomics on two groups of early-stage primary HCC tissues with early (<12 months) and late (>48 months) recurrence after curative resection. We found that transglutaminase 2 (TG2) was upregulated in early-recurrent HCC, suggesting that it may serve as a potential marker for predicting HCC recurrence [4].

TG2 acts as a multifunctional protein, exhibiting cross-linking, GTPase, cell adhesion, protein disulfide isomerase, kinase, and scaffold activities [5]. Given such versatility, TG2 has been found to be involved in several cellular processes, including the regulation of cell growth, differentiation, and apoptosis [6]. The TG2 protein is mainly present in the cytoplasm but also in the cell nucleus, mitochondria, plasma membrane, and extracellular matrix [7]. Interestingly, this multifunctional protein is related to cancer progression, chemoresistance, invasiveness, and metastasis and is upregulated in various cancers [8,9]. However, there are few reports on the role of TG2 in HCC. Sun et al. reported that TG2 was one of the differentially expressed proteins between normal liver (HL-7702) and liver cancer (HepG2 and SK-HEP-1) cell lines, which was validated both in multiple cell lines and in specimens of clinical HCC cases [10]. Yu et al. also reported that TG2 had a tumor-promoting role in HBV-related HCC [11].

However, the detailed relationship between TG2 and HCC remains unclear and requires further investigation. Therefore, in this study, we first knocked down the TG2 gene in the human HCC cell line JHH7. We then analyzed downstream gene expression and cancer cell behavior at the cellular level, further extending our results with clinical data mining.

## 2. Results

### 2.1. TG2 Is Differentially Expressed in HCC Cell Lines

**Figure 1:**
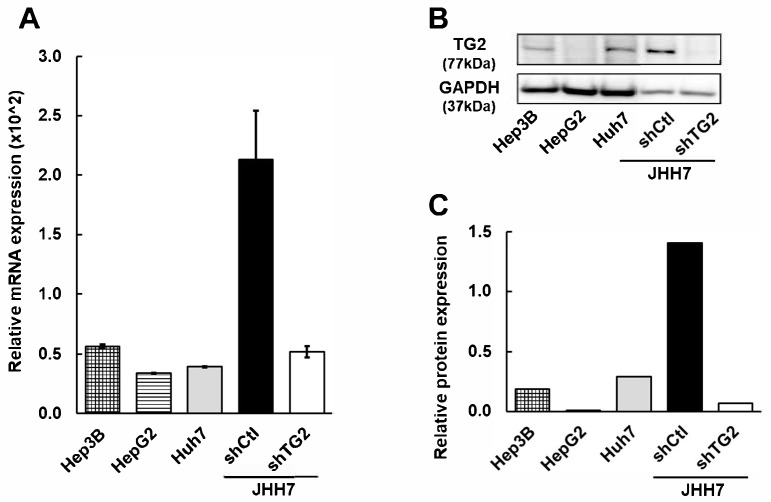
TG2 expression in HCC cell lines. (**A**) Relative mRNA expression level of TG2. The quantity of TG2 mRNA was normalized against that of GAPDH and is shown in bar graph (mean ± SD). (**B**) Western blot image of TG2. GAPDH was used as loading control. (**C**) Relative protein expression of TG2. Intensity of Western blot image of TG2 was normalized against that of GAPDH and was expressed as arbitrary unit.

The expression levels of TG2 were examined in several HCC cell lines (Figure 1). JHH7 cells exhibited higher levels of TG2 than the other HCC cell lines tested (Hep3B, HepG2, and Huh7). TG2-knockdown JHH7 cells (shTG2) and control JHH7 cells (shCtl), prepared as previously described [12], showed differential expression of TG2, confirmed by the qPCR analysis of mRNA and Western blotting (Figure 1). shTG2 reduced TG2 expression in JHH7 cells to levels comparable to those in other HCC cell lines, i.e., Hep3B, HepG2, and Huh7.

### 2.2. TG2 Knockdown Reduces Cell Proliferation but Does Not Affect Migration Ability of JHH7 Cells

**Figure 2:**
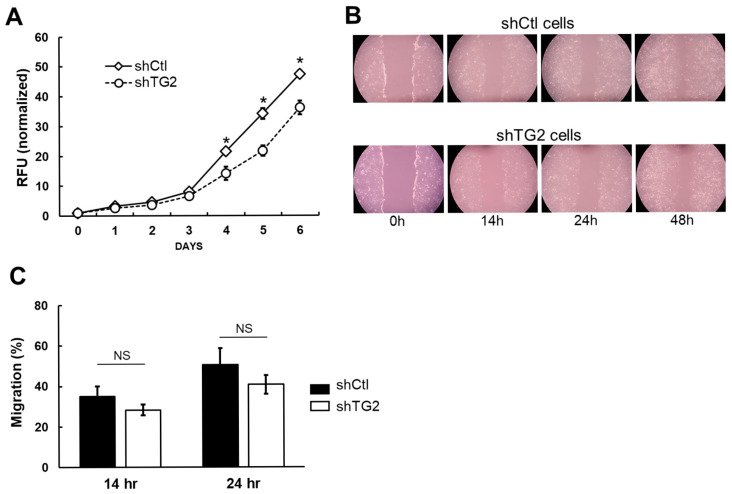
Cell proliferation and migration ability of shCtl and shTG2 cells. (**A**) Cell viability assay was performed with alamarBlue reagent, and the obtained fluorescence values were normalized against those of day 0. Mean ± SD of nine replicates is shown. *, *p* < 0.0001. (**B**) Representative images of in vitro scratch wound healing assay. (**C**) Bar graph of migration rate. Migration rate was analyzed by using MRI Wound Healing Tool from ImageJ MRI Wound Healing Tool (RRID:SCR_025260, version 1.52a) and mean ± SD of three replicates is shown. NS, not significant.

We performed cell viability and migration assays in shCtl and shTG2 cells to investigate the biological role of TG2 expression. The proliferation curve of shTG2 cells was significantly but transiently reduced from day 3 to day 5 compared with shCtl cells (Figure 2A). The wound healing assay for cell migration activity showed no statistically significant difference between the two cell types (Figure 2B,C). Another cell migration assay based on a polycarbonate membrane chamber also failed to show any difference (Supplementary Figure S1).

### 2.3. Gene Expression Signatures of High-TG2 JHH7 Cells

**Figure 3:**
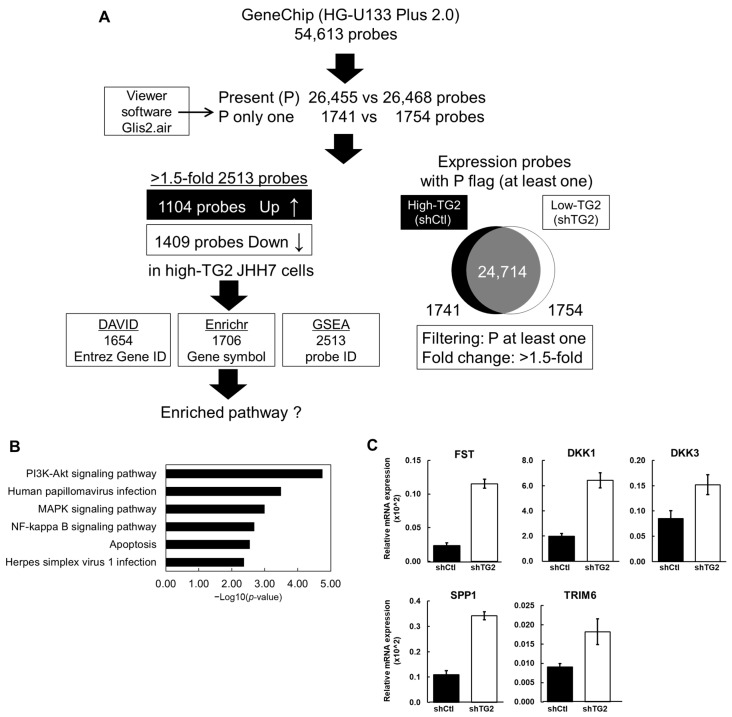
Comprehensive analysis of differentially expressed genes in shCtl and shTG2 cells. (**A**) Flowchart of GeneChip Human Genome U133 Plus 2.0 Array analysis. From 54,613 probe sets, probes present in at least one cell line were chosen for further analysis. More than 1500 differentially expressed genes were selected for pathway analysis. (**B**) Enriched pathways identified by using DAVID platform. The top 6 pathways with *p*-value < 0.05 and FDR *q*-value < 0.25 are displayed as a bar graph, with the x-axis representing −Log10(*p*-value) and the y-axis showing KEGG pathway names. (**C**) mRNA expression of differentially expressed genes in shCtl and shTG2 cells. TaqMan qPCR data (relative expression level, normalized against GAPDH) are shown in a bar graph (mean ± SD).

Next, we carried out the transcriptomic profiling of shCtl and shTG2 cells based on GeneChip analysis to clarify the TG2-dependent gene expression signatures possibly responsible for the TG2-dependent promotion of cell proliferation. A total of 2513 probes out of 54,613 probes exhibited > 1.5-fold change, and of these, 1104 were upregulated, and 1409 were downregulated in shCtl cells compared with shTG2 cells (Figure 3A). With these probe sets (over 1500 genes), we performed pathway analyses by using three different methods (DAVID, GSEA, and Enrichr analysis). Six enriched pathways were found by using the Database for Annotation, Visualization, and Integrated Discovery (DAVID) platform with a cut-off *p*-value < 0.05 and a false discovery rate (FDR) *q*-value < 0.25 (Figure 3B). Among the six pathways identified with DAVID, the PI3K-Akt signaling pathway, which showed the most significant enrichment (*p*-value < 0.0001), was also confirmed with Enrichr (adjusted *p*-value: 0.045).

Following this, we focused on individual genes related to the tumor phenotype (Table 1). The mRNA expression of these genes was confirmed by using the qPCR method. The mRNA levels of FST, DKK1, DKK3, SPP1, and TRIM6 were decreased in shCtl cells compared with shTG2 cells (Figure 3C).

### 2.4. TG2 Is Induced by TGF-β1 but Is Not a Key Player in the Epithelial–Mesenchymal Transition (EMT)

**Figure 4:**
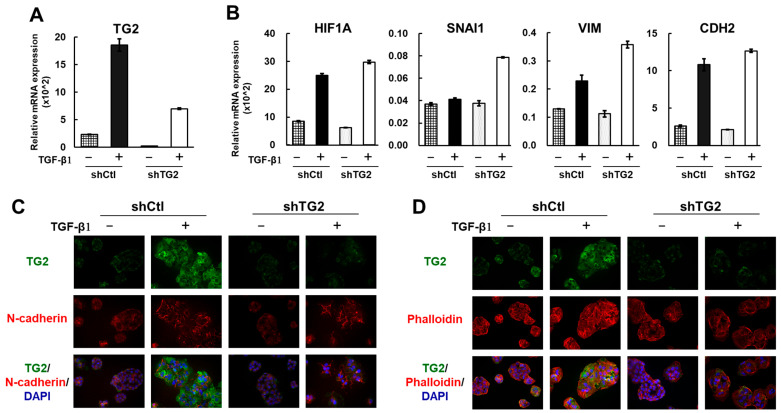
mRNA and protein expression of TGF-β1-induced TG2 and EMT-related genes. (**A**) mRNA expression of TG2 gene in shCtl and shTG2 cells in absence (−) or presence (+) of TGF-β1. Quantity of TG2 mRNA normalized against that of GAPDH is shown in bar graph (mean ± SD). (**B**) mRNA expression of EMT-related genes in shCtl and shTG2 cells without (−) or with (+) TGF-β1. Quantity of mRNAs was normalized against GAPDH and is shown in bar graph (mean ± SD). (**C**) Representative images of immunofluorescence for TG2 (green) and N-cadherin (red) without (−) or with (+) TGF-β1 in shCtl and shTG2 cells. Cell nuclei were visualized by using DAPI. (**D**) Representative phalloidin staining images without (−) or with (+) TGF-β1 in shCtl and shTG2 cells.

It is well known that EMT plays an important role in tumor metastasis and progression. Previously, Kumar et al. reported that TG2 is an important downstream effector of TGF-β1-induced EMT in mammary epithelial cells [21]. Thus, we evaluated EMT-related gene expression in shCtl and shTG2 cells with or without TGF-β1 addition to clarify the association of TG2 with EMT in HCC-derived JHH7 cells. TG2 expression was strongly induced by TGF-β1 treatment (10 ng/mL for 2.5 days) in the shCtl cells, but TG2 was also moderately induced in the knockdown cells. However, there was still enough difference in the expression of TG2 between shCtl and shTG2 cells under TGF-β1 treatment (Figure 4A).

Thus, we anticipated that the change in EMT-related genes induced by TG2 and TGF-β1 could be evaluated, and we determined the expression of these genes under this condition. The mRNA quantitative analysis indicated that the expression of the EMT inducer HIF1A was strongly upregulated by TGF-β1 treatment in both shCtl and shTG2 cells. As a result, the EMT markers vimentin (VIM) and N-cadherin (CDH2) were increased by TGF-β1 with no apparent difference between shCtl and shTG2 cells (Figure 4B,C). In addition, the transcription factor SNAI1 was not induced by TGF-β1 treatment in shCtl cells (Figure 4B).

We also visualized actin fiber formation by using phalloidin staining. Before TGF-β1 treatment, actin fibers were almost absent in both shCtl and shTG2 cells but were primarily observed in the membrane and as slightly granular patterns in the cytoplasm. After TGF-β1 treatment, actin stress fiber formation was observed in shCtl cells, but shTG2 cells also displayed a similar staining pattern (Figure 4D). These results suggest that EMT-related gene expression is independent of TG2 expression in the absence of TGF-β1 and that stimulation by TGF-β1 may also occur in a TG2 dose-independent manner. Thus, TG2’s role in EMT may be small.

### 2.5. TCGA Cohort Study on Characteristics of TG2 and TGF-β1 in Clinical HCC

**Figure 5:**
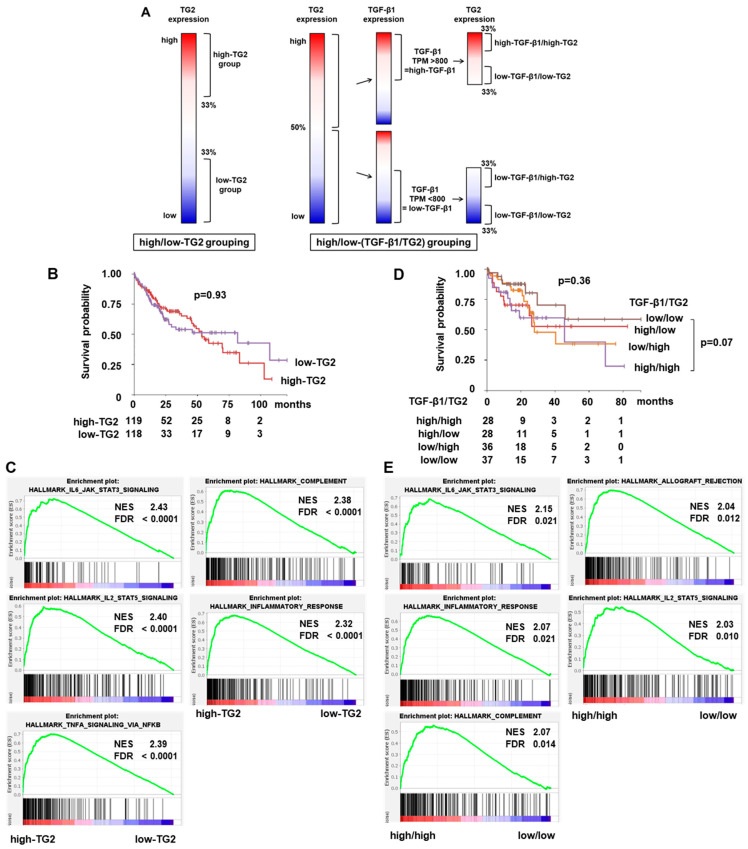
Clinical prognosis and pathway analysis of high-TG2 HCC and high-TGF-β1/high-TG2 HCC in TCGA cohort. (**A**) Schematic illustration showing classification of HCC cases. In total, 366 HCC cases were classified into groups as depicted based on gene expression data from TCGA database. (**B**) Kaplan–Meier curve of OS rate between high-TG2 (*n* = 119) and low-TG2 (*n* = 118) HCC groups. (**C**) GSEA between high-TG2 (*n* = 120) and low-TG2 (*n* = 120) HCC groups. Top five gene sets were determined according to FWER *p*-values. (**D**) Kaplan–Meier curve of OS rate among high-TGF-β1/high-TG2 (*n* = 28), high-TGF-β1/low-TG2 (*n* = 28), low-TGF-β1/high-TG2 (*n* = 36), and low-TGF-β1/low-TG2 (*n* = 37) HCC groups. (**E**) GSEA between high-TGF-β1/high-TG2 (*n* = 28) and low-TGF-β1/low-TG2 (*n* = 38) HCC groups. Top five gene sets were determined according to FWER *p*-values.

We examined TG2 mRNA expression in 366 HCC cases from The Cancer Genome Atlas (TCGA) in relation to the clinical prognosis and specific signal pathways. The two groups of high-TG2 and low-TG2 HCC were determined according to the expression level of TG2, corresponding to those of the shCtl and shTG2 cells in this study: the top 33% and bottom 33% were classified as the high-TG2 and low-TG2 groups, respectively (Figure 5A). There was no difference in the overall survival (OS) between the high-TG2 and low-TG2 HCC groups (Figure 5B), nor in vascular invasion and the tumor stage (Supplementary Table S1). The Gene Set Enrichment Analysis (GSEA) demonstrated that the gene sets involved in inflammation signaling were enriched in the high-TG2 HCC group: IL6-JAK-STAT3 signaling, IL2-STAT5 signaling, the inflammatory response, and TNFα signaling via NFKB (Figure 5C).

We also examined whether TGF-β1/TG2 mutual activation was associated with the prognosis and signal pathway of clinical HCC by using the TCGA cohort. We divided the HCC patients into the four groups of high-TGF-β1/high-TG2, high-TGF-β1/low-TG2, low-TGF-β1/high-TG2, and low-TGF-β1/low-TG2 (Figure 5A). There was no difference in overall survival among the four groups. However, survival in the two groups of high-TGF-β1/high-TG2 and low-TGF-β1/low-TG2 tended to be different. Interestingly, the OS curves show a particular pattern: patients with low-TGF-β1 HCC were likely to experience early-stage survival within 20 months, whereas those with low-TG2 HCC were likely to experience late-stage survival (Figure 5D). The GSEA performed on the two groups of high-TGF-β1/high-TG2 and low-TGF-β1/low-TG2 demonstrated the enrichment of similar gene sets to those described above: four of the top five gene sets ranked by family-wise error rate (FWER) *p*-value were identical in the two comparisons (Figure 5C,E). Furthermore, seven gene sets (including apoptosis and KRAS signaling up) of the top ten enriched in high-TG2 HCC overlapped with all statistically significant gene sets enriched in the high-TGF-β1/high-TG2 HCC group (FWER *p*-value < 0.05). All of these gene sets are related to inflammatory or immune responses. Thus, the inflammatory response is characteristic of high-TG2 HCC, likely mediated by the interaction of inflammatory cells such as macrophages in the high-TGF-β1 tumor microenvironment (TME).

## 3. Discussion

We demonstrate, in this study, that TG2 functions as a promoter for cell proliferation but not for cell migration ability in high-TG2 HCC cells (Figure 2) and suggest that the PI3K-Akt signaling pathway (Figure 3B) and the Wnt/β-catenin signaling pathway are potentially involved in the promotion of cell proliferation (Table 1). Recently, the loss of function of TG2 has been reported to suppress the expression of stemness-related genes and spheroid proliferation and induce the cell death of the EpCAM-positive liver CSC subpopulation in HCC cells [22]. Thus, TG2 promotes cell viability and proliferation. The pathway analysis of the differential gene expression profiles between TG2-knockdown and control cells based on multiple platforms, i.e., DAVID and Enrichr, output the PI3K-Akt signaling pathway (Figure 3B). It is already known that TG2 inhibits PTEN, a negative regulator of the PI3K-Akt signaling pathway, leading to the activation of this pathway in acute promyelocytic leukemia cells and pancreatic cancer cells [23,24]. Although the protein expression or phosphorylation status of key regulators such as PTEN and Akt remains to be examined in this study, TG2 may alter several genes associated with this pathway at the mRNA level, leading to its involvement in cell proliferation.

What is the role of TG2 mRNA expression in clinical HCC samples? The present TCGA cohort study on clinical HCC tissues demonstrates that gene sets related to inflammatory signaling were most prominently enriched in the high-TG2 group (Figure 5C). The similar signaling pathways related to inflammation (virus infection) were also found in the top six enriched pathways identified in HCC cells in vitro (Figure 3B). These TG2-associated signaling pathways may be enhanced due to the presence of stromal cells (macrophages, fibroblasts, etc.) in clinical HCC tissues. TG2 expression is probably closely associated with the interaction of TME in HCC tissues, which is not reflected in cultured HCC cells. Moreover, TG2 mRNA expression alone is not a poor prognostic biomarker of HCC (Figure 5B). HCC is a heterogeneous disease with various etiological factors. S. Shimada et al. classified this type of cancer into three subtypes based on a comprehensive molecular evaluation and clinical features: (1) mitogenic, (2) CTNNB1-mutated, and (3) metabolic disease-associated tumors [25]. Given the molecular and etiological diversity of HCC, it is possible that the prognostic impact of TG2 becomes more evident when focusing on specific subtypes. Indeed, recently, R. Dong et al. demonstrated that high TG2 expression is predictive of a poor prognosis in the form of HCC whose etiological background includes hepatitis B virus infection [26]. These findings highlight the importance of evaluating TG2-related malignancy in a subtype- or context-dependent manner.

It is interesting to note that DKK1, a negative regulator of Wnt signaling, was downregulated in shCtl cells (Table 1 and Figure 3C). DKK1 directly interacts with LRP6, which is a coreceptor of Wnt, and antagonizes the canonical Wnt/β-catenin signaling pathway [15]. TG2 is a novel activator of the canonical LRP6/β-catenin signaling pathway through the cross-linking of the LRP6 receptors [27]. It is well known that in HCC tissues, the Wnt/β-catenin signaling pathway is frequently upregulated and that β-catenin may promote tumor proliferation and progression [28]. Although it remains to be examined whether shTG2 cells actually block Wnt signaling, our data suggest that high-TG2 HCC cells may be more susceptible to Wnt/β-catenin signaling and that this might be associated with their higher viability compared with shTG2 cells.

TG2, on the other hand, is a critical contributor to the EMT in various cancer cells [21,29,30]. The EMT phenotype, however, was not suppressed in shTG2 cells even after TGF-β1 administration (Figure 4). Previously, we reported that TG2 mRNA levels were strongly correlated with TGFB1 but also positively correlated with CDH1 (E-cadherin) mRNA in human HCC tissues [4]. These observations suggest that TG2 may contribute to cancer malignant transformation in an EMT-independent manner in HCC. However, TG2 expression was markedly stimulated by TGF-β1, which is an interesting issue related to the TME effect on tumor cells. Recently, the importance of the TME has been well investigated. TME affects the formation, progression, angiogenesis, chemoresistance, and metastasis of cancer [31,32,33,34]. Moreover, TG2 is a “key modulating player” in the TME [35]; heparan sulfate proteoglycans transport TG2 onto the cell surface and into the ECM [36], and latent TGF-β secreted from cells forms complexes with ECM proteins such as latent TGF-β-binding protein 1 (LTBP1); then, TG2 cross-linking LTBP1 to fibrillin increases TGF-β activation [37]. Thus, TG2 and TGF-β mutually affect their expression and activity. Activated TGF-β induces cancer-associated fibroblast (CAF) activation and the malignant progression of cancers [38]. In our previous study, TG2-positive HCC cells tended to be adjacent to fibrous stroma [4]. Thus, secreted humoral factors such as TGF-β may affect TG2 activities, and the cross-talk between TG2-expressing cancer cells and non-tumor cells (immune cells, fibroblasts, etc.) may be involved in the HCC pathogenesis. The present TCGA cohort study on the TGF-β1/TG2 mutual activation of HCC suggests that TGF-β1 is a poor prognostic factor for early-stage survival, as is TG2 for late-stage survival (Figure 5D). The GSEA results of the high-TGF-β1/high-TG2 and low-TGF-β1/low-TG2 HCC groups also highlighted inflammation signaling and response, suggesting that TGF-β1-dependent TG2 activation overly enhances the inflammatory response, which probably plays a role in the clinical outcome. The significance of TG2-expressing cancer cells in the early-recurrence HCC group should be further investigated by focusing on the communication between HCC and the TME, as well as on the signaling pathways that change due to cancer–stromal interactions, such as the Wnt/β-catenin signaling and TGF-β signaling pathways. The co-culture of HCC cells (shTG2 and shCtl) with TME cells (macrophages, fibroblasts, etc.), for example, could be useful for an in vitro investigation of the molecular mechanism in HCC malignancy driven by TG2.

## 4. Materials and Methods

### 4.1. HCC Cell Lines and Cell Culture

Three HCC cell lines, Hep3B, Huh7, and JHH7, and one hepatoblastoma cell line, HepG2, were used for this study. Each cell line was cultured in low-glucose DMEM (FUJIFILM Wako Pure Chemical Corporation, Osaka, Japan) supplemented with heat-inactivated 10% fetal bovine serum (JRH Bioscience, Lenexa, KS, USA), 1000 U/mL penicillin, and 100 μg/mL streptomycin (Nacalai Tesque, Kyoto, Japan) and maintained in a 5% CO_2_ humidified incubator at 37 °C. JHH7 cells expressing a small hairpin RNA control (shCtl cells, i.e., high-TG2 cells) or those expressing a small hairpin RNA to knock down TG2 expression (shTG2 cells, i.e., low-TG2 cells) were obtained and maintained as previously described [12].

### 4.2. In Vitro Cell Proliferation Assay

alamarBlue Cell Viability Reagent (ThermoFisher Scientific, Waltham, MA, USA) was used to measure the proliferation activity of the shCtl and shTG2 cell lines according to the product manual. A total of 1000 cells per well were seeded in a 96-well plate for cell culture. One plate for each time point was prepared, and the experiment was performed for 6 days in nine replicates per cell line per time point. At each time point, the cells were incubated with alamarBlue reagent for 4 h at 37 °C; then, fluorescence signals were measured at an excitation wavelength of 544 nm and an emission wavelength of 590 nm by using a FlexStation 3 instrument (Molecular Devices, San Jose, CA, USA) and SoftMaxPro software (v.5.4.5).

### 4.3. In Vitro Cell Migration Assay

The wound healing assay was performed as described by Liang et al. [39]. Briefly, 5 × 10^5^ cells (shCtl and shTG2) were seeded into six-well plates in low-glucose DMEM with 1% FBS and incubated until monolayer formation could be observed (48 h). When the cells reached 100% confluence, a sterile P200 pipette tip was used to incise a wound in the cell monolayer, and the debris was removed by gently washing with medium without FBS and then incubated in DMEM with 1% FBS. The resulting gap was examined at 0, 14, 24, and 48 h with a microscope. Several photos were taken at such times and then analyzed by using the Image J MRI Wound Healing Tool (RRID:SCR_025260, version 1.52a https://github.com/MontpellierRessourcesImagerie/imagej_macros_and_scripts/wiki/Wound-Healing-Tool, accessed on 23 March 2019). The CytoSelect 96-Well Cell Migration assay (Cell Biolabs, San Diego, CA, USA) was also performed according to the manufacturer’s protocol by using various concentrations of FBS (10%, 1%, and 0.1%) in culture medium and various cell numbers (100,000, 50,000, 25,000, and 12,500 cells per well).

### 4.4. GeneChip Transcriptome Analysis

Total RNA from shCtl and shTG2 cells was used for gene expression profiling. GeneChip assays were performed by using the GeneChip 3′ IVT PLUS Reagent Kit and the Affymetrix Human Genome U133 Plus 2.0 Array (HG-U133 Plus 2.0) (ThermoFisher Scientific), according to the manufacturers’ instructions. Data were analyzed by using MAS5.0 with default settings, and fold changes were calculated with Glis2 Viewer software.

Database for Annotation, Visualization, and Integrated Discovery (DAVID) (https://david.ncifcrf.gov/) [40,41], Gene Set Enrichment Analysis (GSEA; version 4.3.3) [42,43], and the Enrichr database (https://maayanlab.cloud/Enrichr/, accessed on 23 March 2019) [44,45,46] were used for data analysis and interpretation.

### 4.5. Quantitative PCR (qPCR) Assay of mRNA

A total of 1 µg of total RNA was used for cDNA synthesis by using the ReverTra Ace qPCR RT Master Mix with gDNA Remover Kit (TOYOBO, Osaka, Japan). The expression levels of the target gene mRNAs were determined with qPCR by using 10 ng of cDNA and the TaqMan Fast Advanced Master Mix (ThermoFisher Scientific), following the manufacturer’s protocol, with a Step One Plus Real-Time PCR System (ThermoFisher Scientific). The TaqMan assay IDs used in this study are as follows: GAPDH (Hs02786624_g1) as a housekeeping gene, TGM2 (Hs01096681_m1), FST (Hs00246256_m1), DKK1 (Hs00183740_m1), DKK3 (Hs00951307_m1), SPP1 (Hs00959010_m1), TRIM6 (Hs04194831_s1), HIF1A (Hs00153153_m1), SNAI1 (Hs00195591_m1), VIM (Hs00958111_m1), and CDH2 (Hs00983056_m1). The quantity of mRNA was normalized to GAPDH by using the relative quantitation (ΔCt) method.

### 4.6. Western Blot Analysis

Cells from each HCC cell line were cultured in 6-well plates (~80% confluence) and then used for total protein extraction with RIPA cell lysis buffer supplemented with protease and phosphatase inhibitors. The protein concentration was determined with the Pierce bicinchoninic acid (BCA) Protein Assay Kit (ThermoFisher Scientific). A total of 50 µg of total protein was separated by using 3–8% Tris-Acetate gels from the NuPAGE system (ThermoFisher Scientific) and blotted onto nitrocellulose membranes (BioRad, Hercules, CA, USA). The membranes were incubated in blocking solution containing 5% bovine serum albumin in 1× PBS for 1 h and subsequently incubated with primary antibodies overnight at 4 °C. The primary antibodies were as follows: anti-human TG2 (1/10000; rabbit monoclonal, ab109200; Abcam, Cambridge, UK) and anti-human GAPDH (1/10000; rabbit polyclonal, ab37168; Abcam), which was used as an internal reference. Then, the membranes were incubated with horseradish peroxidase-conjugated secondary antibody (1/1000; Dako polyclonal swine anti-rabbit, P0217; Agilent Technologies, Santa Clara, CA, USA). The target protein was detected by using ECL Prime Western Blotting Detection Reagent (Cytiva, Tokyo, Japan) and a chemiluminescence detection system, ImageQuant LAS 500 (Cytiva).

### 4.7. Immunofluorescence Analysis

The cells were seeded on glass slides and treated with 10 ng/mL recombinant human TGF-β1 (PeproTech, Cranbury, NJ, USA) for 2.5 days. Immunofluorescence staining was performed as described previously [47]. Briefly, the cells on glass slides were fixed with 4% paraformaldehyde and blocked in 5% skim milk; then, they were incubated with primary antibodies (mouse anti-TG2, ab2386 (Abcam); mouse anti-N-cadherin (BD Biosciences, San Jose, CA, USA); rabbit anti-β-Catenin, #8480 (Cell Signaling Technology, Danvers, MA, USA)). Fluorescent dye-conjugated antibodies (Alexa488-conjugated or Alexa546-conjugated anti-mouse or rabbit IgG; ThermoFisher Scientific) were used as secondary antibodies. The cell specimens were also stained with rhodamine phalloidin (Cytoskeleton, Inc., Denver, CO, USA). The nuclei were stained with 4′,6-diamidino-2-phenylindole (DAPI; ThermoFisher Scientific). Cellular fluorescence signals were detected by using a fluorescence microscope (BZ-X800 Analyzer) and BZ-Viewer software (Keyence, Osaka, Japan).

### 4.8. Statistical Analysis

Quantitative data are expressed as the means ± SDs of at least three replicates. Statistical comparisons were assessed with a two-tailed Student’s *t* test, and differences were considered significant at *p* < 0.05. The statistical analysis of data was performed by using GraphPad Prism software (version 5.01; GraphPad Software Inc., San Diego, CA, USA).

### 4.9. Data Mining from Clinical HCC Database

The RNA-seq data of the Liver Hepatocellular Carcinoma (TCGA, PanCancer Atlas) cohort were obtained from cBioPortal (https://www.cbioportal.org/, accessed on 15 January 2025). The Kaplan–Meier survival analysis was performed by using the Xena platform [48]. The GSEA was conducted by using GSEA 4.3.3, and the other statistical analyses were conducted by using JASP computer software (version 0.19.3; JASP Team, 2024; https://jasp-stats.org/).

## Figures and Tables

**Table 1:** Differentially expressed genes in shCtl cells and shTG2 cells.

Gene Name	Gene Symbol	GeneChip			Gene Functions Relevant to Cancer	Ref.
		Fold Change	shCtl Signal	shTG2 Signal		
Follistatin	FST	0.442	50.4	114.0	Tumor progression through antagonism of TGF-β family members	[13]
Dickkopf WNT signaling pathway inhibitor 1	DKK1	0.395	1313.3	3325.2	Negative regulator of Wnt signaling targeting β-catenin/TCF pathway	[14,15]
Dickkopf WNT signaling pathway inhibitor 3	DKK3	0.557	89.9	161.3	HCC suppressor and Wnt modulator	[16,17]
Secreted phosphoprotein 1	SPP1	0.252	64.0	254.5	Tumor promoter through interaction with carcinogenic genes and facilitator of immune cell infiltration	[18]
Tripartite motif-containing 6	TRIM6	0.258	72.7	281.4	Potential prognostic marker for colorectal cancer and hepatocellular carcinoma	[19,20]

The differentially expressed genes identified with GeneChip analysis and validated with qPCR were summarized.

## Data Availability

The original contributions presented in this study are provided within the article. Further data will be shared upon request.

## Supplementary Materials

The following supporting information can be downloaded at: https://www.mdpi.com/article/10.3390/ijms26125497/s1.

## Abbreviations

The following abbreviations are used in this manuscript:

TG2	Transglutaminase 2
HCC	Hepatocellular carcinoma
EMT	Epithelial–mesenchymal transition
TGF-β1	Transforming growth factor-beta 1
DAVID	Database for annotation, visualization, and integrated discovery
GSEA	Gene set enrichment analysis
FDR	False discovery rate
TCGA	The cancer genome atlas
OS	Overall survival
NES	Normalized enrichment score
FWER	Family-wise error rate
