# Supplementary material for: Transglutaminase 2 Stimulates Cell Proliferation and Modulates Transforming Growth Factor-Beta Signaling Pathway Independently of Epithelial–Mesenchymal Transition in Hepatocellular Carcinoma Cells
Source: Int J Mol Sci. 2025 Jun 8;26(12):5497. doi: 10.3390/ijms26125497 (PMC12192954; doi:10.3390/ijms26125497)
Supplement: Supplementary file 1 [file ijms-26-05497-s001.zip › ijms-3625572-supplementary.pdf]

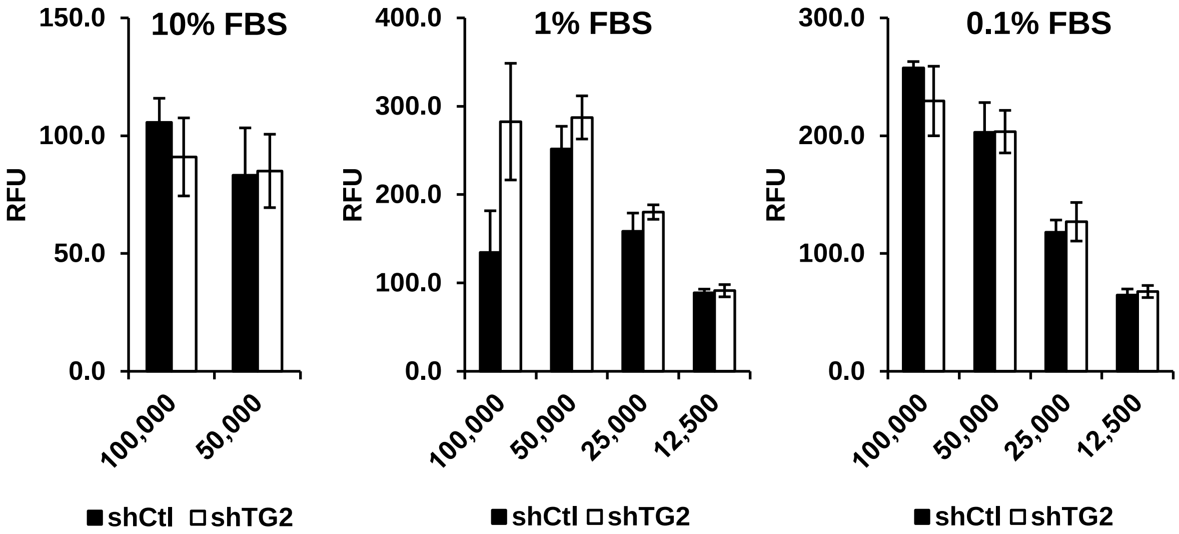

Figure S1. Cell migration ability of shCtl and shTG2 cells assessed by using the CytoSelect 96-Well Cell Migration Assay Kit. Cell migration was evaluated under various concentrations of FBS (10%, 1%, and 0.1%) and different cell seeding densities (100,000; 50,000; 25,000; and 12,500 cells per well). Mean ± SD are shown.

Table S1. Association between TG2 expression and clinicopathological features assessed by chi-squared test.

Clinicopathological features	Low-TG2	High-TG2	Total	*p*-value
**Vascular invasion**				
−	64	63	127	
+	35	40	75	0.609
Total	99	103	202	
**Tumor stage**				
Stage I	53	56	109	
Stage II	30	22	52	0.533
≧Stage III	30	31	61	
Total	113	109	222	
